# Addition of Butyric Acid and Lauric Acid Glycerides in Nursery Pig Feed to Replace Conventional Growth Promoters

**DOI:** 10.3390/ani14081174

**Published:** 2024-04-13

**Authors:** Cássio Antônio Ficagna, Gabriela Miotto Galli, Emerson Zatti, Isadora Zago, Marco Aurélio Fritzen Dias do Amaral, Maksuel Gatto de Vitt, Diovani Paiano, Aleksandro Schafer da Silva

**Affiliations:** 1 Graduate Program and Animal Science, University of Santa Catarina State (UDESC), Rua Beloni Trombeta Zanini, nº 680, Bairro Santo Antônio, Chapecó 89815-630, SC, Brazil; cassioficagna98@gmail.com (C.A.F.); emersonzatti@gmail.com (E.Z.); zagoisadora@gmail.com (I.Z.); marco.afdda@edu.udesc.br (M.A.F.D.d.A.); mak-witt@hotmail.com (M.G.d.V.); diovani.paiano@udesc.br (D.P.); 2 Graduate Program in Animal Science, Federal University of Rio Grande do Sul (UFRGS), Avenida Paulo Gama, nº 110, Farroupilha, Porto Alegre 90010-150, RS, Brazil; gabi-gmg@hotmail.com

**Keywords:** additives, immunity, performance, swine, tributyrin

## Abstract

**Simple Summary:**

Determining whether adding butyric acid and lauric acid glycerides to pigs’ diets would replace the growth promoters was our goal. The additives fulfilled their purpose, resulting in great growth performance and lower therapeutic interventions. The additives increased the immunoglobulins and decreased the acute phase proteins, which reflects a lower inflammatory response.

**Abstract:**

(1) Background: This study determined whether adding butyric acid and lauric acid glycerides in nursing pigs’ feed would improve growth performance, proteinogram, biochemical parameters, and antioxidant status. (2) Methods: Ninety male pigs were divided into five groups with six repetitions per group: NC, negative control (no additive); TRI-BUT, addition of tributyrin in the basal ration; MDT-BUT, addition of mono-, di-, and triglycerides of butyric acid in the basal feed; MDT-LAU, the addition of mono-, di-, and triglycerides of lauric acid in the basal feed; and PC, positive control (addition of gentamicin in the basal feed). (3) Results: PC, TRI-BUT, and MDT-LAU resulted in a high average daily WG from days 1 to 39 (*p* < 0.01). MDT-LAU, MDT-BUT, and PC resulted in a greater feed:gain from days 1 to 39 than the NC (*p* = 0.03). Great concentrations of the gamma globulin fraction in all groups were observed than in the NC (*p* = 0.01). Ceruloplasmin, haptoglobin, and C-reactive protein concentrations were lower in all groups than in the NC (*p* < 0.05). Higher serum glutathione S-transferase activity was observed in the TRI-BUT and MDT-BUT than in the PC (*p* = 0.04). (4) Conclusions: The addition of butyric acid and lauric acid glycerides in the diet of pigs in the nursery phase can replace growth promoters since the products improve the growth performance, reduce acute-phase proteins, and increase gamma globulin concentrations.

## 1. Introduction

Due to several challenges, the nursery phase is one of the most critical phases in a pig’s life. Many of these challenges (i.e., physiological and immunological immaturity) correspond to weaning, with life conditions imposed on the pigs (diet change, separation from the mother, and forming new social groups).

Heritable factors do not primarily influence the survival and performance of pigs in the nursery phase; instead, they are related to environmental factors such as nutrition, health, management, and facilities [1]. The imposed challenges can cause intestinal dysbiosis, which leads to lower performance due to diarrhea and facilitates infections with opportunistic pathogens [2].

Limitations on using antibiotics as growth promoters in animal feed are a consequence of public health policies. The European Union banned the use of antimicrobials as growth promoters in 2006 [3]. Brazil, the fourth largest exporter of pork worldwide, enacted a partial ban on some drugs in 2018–2020 [4,5]. For these reasons, alternatives are sought to replace growth promoters (antibiotics), including organic acids, phytogenics, probiotics, and prebiotics.

Among commercially available additives, we highlight butyric acid, composed of four carbons, and its derivatives, including mono-, di-, and triglycerides. This acid is linked to a glycerol molecule via an ester bond. Furthermore, this acid is an energy source for enterocytes and improves intestinal morphology [6]. Tributyrin comprises three butyrate molecules and is released by intestinal lipase [7]. The mono-, di-, and tri-forms of butyric acid refer to one, two, and three molecules of butyrate attached to glycerol, leading to an enhancement of gut development, control of enteric pathogens, reduction of inflammation, improvement of growth performance (including carcass composition), and modulation of gut microbiota [8]. Tributyrin improved the growth performance of weaned pigs in the same way that it increases protein utilization and synthesis [9].

The mono-, di-, and triglyceride forms of lauric acid are monoesters comprising 12 carbon atoms and characterized by the union between a glycerol molecule and lauric acid. Lauric acid can be found in nature, e.g., in coconut oil and human breast milk in the glycerol monolaurate [10]. It has fungicidal, bactericidal, viricidal [11], and antioxidant activity [12], in addition to improving immunity [13]. Glycerol monolaurate improved the digestion and absorption of nutrients and reduced the colonization of pathogenic bacteria in weaned pigs [14]. There is a lack of literature on how additives can influence serum biomarkers that are related to the immune system and biochemistry systems. Therefore, this study aimed to determine whether adding butyric acid and lauric acid glycerides to nursing pigs’ feed would improve growth performance, proteinogram, serum biochemistry, and antioxidant status.

## 2. Materials and Methods

### 2.1. Additives

The additives tested were based on glycerides: (1) mono-, di-, and triglycerides of butyric acid, (2) tributyrin, (3) mono-, di-, and triglycerides of lauric acid, and the antibiotic gentamicin.

### 2.2. Animals and Experimental Design

We used 90 non-castrated male weaned pigs (Landrace x Large White), 28 days old, with an average weight of 7.5 (±1.1 kg). The pigs were randomly assigned to one of five treatments with six replicates each and three pigs per pen (90 cm × 1.20 m). The treatment groups were as follows: NC, negative control (no additive, only basal feed); TRI-BUT, addition of tributyrin in the basal feed (dose of 2 kg/ton in the pre 1, pre 2 feed; and 1 kg/ton in the starter feed); MDT-BUT, addition of mono-, di-, and triglycerides of butyric acid (dose of 2 kg/ton in the pre 1, pre 2 feed; and 1 kg/ton in the starter feed); MDT-LAU, the addition of mono-, di-, and triglycerides of lauric acid (2 kg/ton in the pre 1, pre 2 feed; and 1 kg/ton in the starter feed); PC, positive control, addition of 0.113 kg/ton of gentamicin in the pre 1, pre 2, and starter feed.

The basal feed used to prepare the experimental diets was formulated based on crushed corn and soybean meal (Table 1), according to the nutritional requirements for pigs described in the Brazilian Tables for Poultry and Swine [15]. The pigs received water and feed ad libitum.

### 2.3. Performance

Pigs were weighed on days 1, 7, 14, and 39 of the experiment using a scale. Weight gain (WG) was determined by the formula = final weight minus the initial weight of the group. Feed intake was calculated as the difference between the feed provided and the leftovers weighed at the end of the period. Feed:gain was calculated by the total amount of feed ingested divided by the live weight of the pigs.

### 2.4. Sample Collection

Veterinary drug interventions were performed whenever the pigs showed diarrhea and signs of apathy, neurological signs, encephalitis, or signs of gastrointestinal or respiratory disease. We treated diarrhea using a protocol based on lincomycin (Linco Spectin^®^—1 mL/10 kg, three doses at 24-h intervals). We treated encephalitis with ampicillin and colistin (Agroplus^®^—1 mL/10 kg, three doses at 24-h intervals). The number of medicated pigs and doses per pig were counted and presented descriptively.

On day 39, we chose one pig per pen randomly for drawn blood—a total of six pigs per treatment; into vacutainer tubes without anticoagulant via the cranial vena cava. This material was centrifuged at 7000 rpm for 10 min, and the serum was separated, collected, and frozen (–20 °C) for biochemical analysis, proteinogram, and antioxidant status.

### 2.5. Serum Biochemistries

Serum concentrations of albumin, total protein, cholesterol, triglycerides, and urea were analyzed using commercial analytical kits (Analisa^®^) and a semi-automatic biochemical analyzer (Bioplus 2000^®^). The serum globulin level was calculated using globulin = total protein—albumin.

### 2.6. Proteinogram

Protein fractionation was performed using cellulose acetate strip electrophoresis in a horizontal cube (Denscan^®^ Electrophoresis System, Labex S/A Indústria e Comércio, Aparecida de Goiânia, Brazil) with Tris-glycine buffer (pH 8.6) as described by Costa et al. [16]. Samples were applied to the strips and run using a constant voltage of 150 V for 25 min. Strips were stained with Ponceau Red for 15 min. The excess stain was removed by washing the strips with 5% acetic acid until the background was clear. Then, strips were fixed in methanol for 30 s and washed for 1 min with a destain solution. Strips were dried at 60 °C for 15 min and read using the Denscan system.

### 2.7. Antioxidant Status

Protein thiols (PSH) were determined by the method described by Sedlak and Lindsay [17]. PSH was measured in the pellet formed by protein precipitated from the resuspended material using a homogenization buffer. Absorbance readings (405 nm) were performed using a spectrofluorometer (Biotek, Synergy HT). Serum glutathione S-transferase (GST) activity was determined according to Habig et al. [18] from the formation rate of dinitrophenyl-S-glutathione at 340 nm in a medium containing 50 mM potassium phosphate, pH 6.5, 1 mM GSH, 1 mM 1-chloro-2,4-dinitrobenzene (CDNB) as substrate and tissue supernatants (approximately 0.045 mg of protein). Lipid peroxidation was determined as thiobarbituric acid reactive substances (TBARS) levels in serum according to the method described by Jentzsch et al. [19]; results were obtained using spectrophotometry at 535 nm.

### 2.8. Statistical Analysis

All data were analyzed using the SAS ‘MIXED procedure’ (SAS Inst. Inc., Cary, NC, USA; version 9.4), with the Satterthwaite approximation to determine denominator degrees of freedom for the fixed effects test. All variables were subjected to the normality test (Shapiro–Wilk). Body weight (BW), weight gain, concentrate intake, and blood date were tested for fixed treatment effect using pen (repetition) as the random effect, as well as treatment × day interaction to BW. Day 1 results were included as an independent covariate. Means were separated using the PDIFF method (*t*-test), and all results were expressed as LSMEANS followed by the standard error of the mean. Significance was defined when *p* ≤ 0.05; a trend was when *p* > 0.05 or ≤0.10.

## 3. Results

### 3.1. Growth Performance

The growth performance results are presented in Table 2. Greater body weight was observed on day 39 of the experiment in the PC, TRI-BUT, and MDT-LAU groups than in the NC (*p* < 0.01). PC, TRI-BUT, and MDT-LAU resulted in a greater average daily weight gain from days 1 to 39 than in the NC (*p* < 0.01). There was a trend toward greater feed intake by pigs in the PC group than the others between days 15 and 39 (*p* = 0.10). There was lower feed:gain from days 1 to 14 in the TRI-BUT and MDT-BUT groups, followed by the PC, than the NC (*p* = 0.05). In the total period (days 1–39), there was a lower feed:gain in the pigs in the MDT-LAU group, followed by the MDT-BUT and PC, than the NC (*p* = 0.03). There was no difference for the other periods (*p* > 0.05).

### 3.2. Serum Biochemistries, Proteinogram, and Antioxidant Status

The results of serum biochemistry, electrophoresis protein profile, and antioxidant status are displayed in Table 3. A greater glucose concentration was observed in the PC and TRI-BUT groups than in the others (*p* = 0.05). Lower levels of triglycerides were observed in the MDT-BUT and MDT-LAU groups, followed by the TRI-BUT, than in the NC (*p* = 0.02). There were greater concentrations of urea and ML in the PC than in the NC (*p* = 0.01). There was no difference in cholesterol, total protein, and albumin (*p* > 0.05). There was a trend toward lower globulin levels in the TRI-BUT and MDT-BUT groups than in the PC group (*p* = 0.08).

There were greater observed concentrations of gamma globulins in all groups than in the NC (*p* = 0.01). The concentrations of ceruloplasmin, haptoglobin, and C-reactive protein were lower in all groups than in the NC (*p* = 0.02; *p* = 0.01; *p* = 0.04). Greater GST activity was observed in the TRI-BUT and MDT-BUT groups than in the PC (*p* = 0.04). However, there was no difference between groups for TBARS and PSH (*p* > 0.05).

### 3.3. Drug Interventions

One pig in the PC group died on the eighth day of the experiment, with clinical neurological signs compatible with rapidly evolving encephalitis, which did not allow antibiotics. The number of pigs medicated with lincomycin due to diarrhea was seven in the PC group, 13 in the NC group, five in the TRI-BUT group, nine in the MDT-BUT group, and eight in the MDL-LAU group. The number of pigs medicated with ampicillin and colistin due to suspected encephalitis was four in the PC group, six in the NC group, three in the TRI-BUT group, five in the MDT-BUT group, and four in the MDT-LAU group. It is essential to highlight that some pigs were medicated with these drugs at different times, and some pigs needed to be medicated more than once due to diarrhea. Therefore, when interpreting the data on the number of doses of lincomycin given during the entire experimental period, it is worth remembering that our protocol provided for three doses in a 24-h interval. The number of pigs medicated according to the groups was as follows: PC, NC, TRI-BUT, MDT-BUT, and MDT-LAU received 30, 60, 18, 27, and 24 doses, respectively.

## 4. Discussion

The group composed of mono-, di-, and triglycerides of lauric acid also showed a higher WG than the NC, similar to the PC. This finding could be because of the ability of the lauric acid molecule to increase villus height and villus:crypt ratio and decrease crypt depth [20]. This finding partially explains the WG, i.e., being due to a greater surface area for the absorption of nutrients and lower energy expenditure with the renewal of intestinal cells. In addition, this molecule has antimicrobial action due to being linked to a glycerol molecule; thus, it can more easily cross the liposoluble membrane of bacteria and release hydrogen ions. The bacterium dies from energy depletion in an attempt to send hydrogen out of the cell.

Tributyrin improved post-weaned pigs’ enzymatic activity of lactase, maltase, lipase, and trypsin [21]. Taherpour et al. [22] observed that broiler chickens’ mono-, di-, and triglyceride forms of butyric acid decreased pathogenic bacteria. Wang et al. [23] reported that adding tributyrin to the diet of weaned piglets increased daily WG, villus size, and villus:crypt ratio and reduced crypt depth, which improved the fecal score. Tugnoli et al. [24] found that tributyrin addition improved the growth performance of pigs, as the additive stimulated the proliferation of intestinal villi. These results might explain the superior performance of pigs from the MDT-BUT and TRI-BUT groups concerning the NC, similar to when using the growth promoter gentamicin.

Lauric acid is transported directly to the liver and metabolized as an energy source in the mitochondria [13], so it can modify lipid metabolism. Adding mono-, di-, and triglycerides of lauric acid decreased serum triglyceride concentrations in our pigs. Saeidi et al. [25] reported a decrease in triglycerides, total cholesterol, and LDL cholesterol and an increase in HDL cholesterol when supplementing quail with medium-chain fatty acids; nevertheless, the mechanisms of how these molecules alter lipid metabolism are uncertain. Urea is a waste product produced in the liver from the breakdown of proteins by the body, and higher levels in the blood are related to kidney or liver problems [9]. We found a great daily WG in pigs in the MDT-LAU group associated with higher serum levels of urea; however, the values of this variable were within the normal range for the species and nursery phase.

Pigs in groups fed with butyric acid or lauric acid glycerides had lower serum triglyceride concentrations than the negative control. The cause of the occurrence is related to the lower lipoprotein lipase concentration in the jejunum, liver, and adipocytes, which means there was a reduction in lipolysis [26]. Glucose is used by cells as a source of energy; therefore, it reflects the animals’ physiological state [27]. There was an increase in glucose concentration in the group that received tributyrin. This increase may be related to hyperglycemia [28], observed in the current study discreetly, but higher than the other treatments.

The body has enzymatic and non-enzymatic antioxidant defense systems to combat excess free radicals. GST is a metabolic enzyme crucial to the organism’s detoxification [29]. In addition, it participates in the synthesis of steroids; in cases of oxidative stress, its levels are often reduced [30]. Butyric acid glycerides caused an increase in GST activity in the present study, which may have occurred to combat a possible excess of reactive oxygen species. Lan et al. [31] found that the supplementation of 1200 mg/kg of butyric acid in broiler chickens increased the activity of the antioxidant enzymes superoxide dismutase and glutathione peroxidase and decreased malondialdehyde levels.

Gamma globulin concentration encompasses all immunoglobulins (IgA, IgG, IgM, and IgE). Our findings suggest that the antimicrobial and the glycerides efficiently reduce the immune or inflammatory response by increasing the gamma globulin levels that act against foreign organisms entering the body. Haptoglobin and C-reactive protein are acute-phase proteins [32] and have their concentrations altered when innate immunity is activated [33]. Ceruloplasmin is an acute-phase protein that carries copper from the liver to other tissue cells [34]. Acute-phase proteins are produced and secreted by hepatocytes and extrahepatic tissues such as epithelial cells, endoepithelial cells, and connective tissue [35]. Therefore, the increase in these proteins indicates inflammation and activation of the immune response, which leads to energy expenditure. Our findings suggest that the additives could mitigate these effects.

## 5. Conclusions

The use of butyric acid and lauric acid glycerides in the diet of pigs in the nursery phase has the potential to replace growth promoters, as the additives fulfilled their purpose in improving growth performance and reducing therapeutic interventions; they also modulated protein, energy, and lipid metabolism. Glyceride forms of butyric acid stimulated GST, an enzyme essential for liver protection. Finally, the additives increased the concentration of immunoglobulins and decreased the concentration of acute-phase proteins, which reflect a reduced inflammatory response.

## Figures and Tables

**Table 1:** Ingredients and calculated chemical composition of diets.

Ingredients	Pre-Nursery I	Pre-Nursery II	Initial
Corn Grain 7.8% CB	228	323	407
Alpha Extruded Corn	200	200	200
Soy Meal 45% CB	210	255	260
Deactivated Whole Soybean	80	60	50
Prot. Conc. Soy X-Soy 200	40	10	-
Dehydrated Egg Flour	20	5.0	-
Whey—Relat	140	70	-
Crystal Sugar	30	20	10
Limestone 38% Ca	8.7	7.5	8.4
Dicalcium phosphate—Phosbic	10	9.9	11
Sodium Bicarbonate	-	-	5.62
Refined Salt	5.4	5.5	3.1
L-Lysine 98.5%/78	4.64	4.86	6.44
DL-Methionine 99%	2.29	2.05	2.42
L-Threonine 98.5%	4.16	4.03	4.82
L-Tryptophan 98%	0.41	0.45	0.7
L-Isoleucine	-	-	0.38
L-Valine	1.22	1.20	1.93
Bewi-Spray 99L	4.82	12.3	20
PX VITAMIN	3.0	3.0	3.0
PX MINERAL	3.0	3.0	3.0
Vit. E 50%	0.1	0.1	0.1
Zinc Oxide 75% Zn	3.5	2.5	1.0
Sugarcap	0.2	0.2	0.15
Banox	0.4	0.4	0.4
Total weight	1000	1000	1000
	Nutritional Levels		
Moisture %	8.91	9.84	10.5
Crude Protein %	21.4	20.1	19.3
Milk Protein %	1.68	0.84	-
Total Lactose %	10.5	5.25	-
Amide %	28.5	34.6	40.0
Ethereal Extract %	5.11	5.22	5.80
Crude Fiber %	2.44	2.60	2.69
Lysine Dig Sui %	1.45	1.34	1.36
Methionine Dig Sui %	0.52	0.47	0.49

**Table 2:** Body weight (kg), average daily gain (kg), feed intake (kg/day), and feed:gain (kg/kg) of pigs fed formulations derived from butyric acid and lauric acid.

	Treatment ^1^						*p*-Values ^2^	
	PC	NC	TRI-BUT	MDT-BUT	MDT-LAU	SEM	Treat	Treat × Day
Body weight (kg)							0.12	<0.01
d 1	8.15	8.15	8.16	8.16	8.15	0.21		
d 7	9.3	9.41	9.34	9.3	9.25	0.18		
d 14	11.9	11.9	12.2	12.1	12.3	0.19		
d 39	24.8 a	23.3 b	24.3 a	24.0 ab	24.6 a	0.16		
Daily weight gain (kg)								
d 1–7	0.17	0.18	0.17	0.16	0.16	0.02	0.80	-
d 8–14	0.38	0.36	0.36	0.36	0.4	0.01	0.21	-
d 15–39	0.51	0.46	0.46	0.47	0.49	0.02	0.14	-
d 1–14	0.27	0.27	0.28	0.28	0.29	0.01	0.16	-
d 1–39	0.42 A	0.38 B	0.41 A	0.40 AB	0.42 A	0.01	<0.01	-
Feed intake (kg/day)								
d 1–7	0.26	0.27	0.25	0.26	0.27	0.02	0.95	-
d 8–14	0.51	0.51	0.49	0.48	0.57	0.03	0.30	-
d 15–39	0.92 A	0.86 B	0.88 B	0.87 B	0.86 B	0.02	0.10	-
d 1–14	0.39	0.39	0.37	0.37	0.42	0.02	0.57	-
d 1–39	0.73	0.7	0.7	0.69	0.7	0.02	0.51	-
Feed:gain (kg/kg)								
d 1–7	1.66	1.53	1.49	1.7	1.9	0.17	0.40	-
d 8–14	1.35	1.42	1.37	1.34	1.4	0.04	0.32	-
d 15–39	1.79	1.9	1.93	1.85	1.74	0.07	0.17	-
d 1–14	1.44 B	1.49 A	1.35 C	1.35 C	1.47 AB	0.04	0.05	-
d 1–39	1.73 BC	1.85 A	1.79 AB	1.72 BC	1.65 C	0.05	0.03	-

^1^ Positive control (PC), negative control (NC), tributyrin (TRI-BUT), mono-, di-, triglycerides of butyric acid (MDT-BUT) and mono-, di-, triglycerides of lauric acid (MDT-LAU). ^2^ Different Lowercase (a, b) letters on the same line indicate interaction between treatment × day; and different capital letters (A, B, C) indicate treatment effect in the same line.

**Table 3:** Serum biochemistry and antioxidant status of pigs fed formulations derived from butyric acid and monolaurin.

Items ^1^	Treatment ^1^					SEM	*p*-Values ^2^
	PC	NC	TRI-BUT	MDT-BUT	MDT-LAU		
Serum biochemistry							
Glucose (mg//dL)	116 ^ab^	106 ^b^	133 ^a^	101 ^b^	102 ^b^	3.98	0.05
Cholesterol (mg/dL)	120	111	132	114	122	4.74	0.53
Total protein (g/dL)	8.02	7.61	6.94	6.68	7.55	0.69	0.25
Albumin (g/dL)	3.04	3.18	3.2	3.12	3.08	0.09	0.84
Triglycerides (mg/dL)	48.8 ^ab^	57.1 ^a^	43.8 ^b^	34.2 ^c^	35.6 ^c^	2.85	0.02
Urea (mg/dL)	32.0 ^a^	18.0 ^b^	24.8 ^ab^	25.0 ^ab^	32.1 ^a^	2.36	0.01
Globulin (g/dL)	4.97 ^a^	4.43 ^ab^	3.74 ^b^	3.56 ^b^	4.46 ^ab^	0.15	0.08
Proteinogram							
Gamma globulin (g/dL)	0.95 ^a^	0.64 ^b^	0.97 ^a^	0.85 ^a^	0.95 ^a^	0.2	0.01
Ceruloplasmin (g/dL)	0.39 ^b^	0.78 ^a^	0.34 ^b^	0.31 ^b^	0.36 ^b^	0.07	0.02
Haptoglobin (g/dL)	0.45 ^b^	0.64 ^a^	0.46 ^b^	0.42 ^b^	0.45 ^b^	0.06	0.01
C-reactive protein (g/dL)	0.25 ^b^	0.41 ^a^	0.24 ^b^	0.22 ^b^	0.27 ^b^	0.03	0.04
Oxidative status							
TBARS (nmol MDA/mg protein)	5.62	4.98	4.53	6.04	6.41	1.03	0.39
GST (µmol CDNB/min/mg protein)	454 ^b^	477 ^b^	573 ^a^	557 ^a^	488 ^ab^	6.52	0.04
Protein thiols (nmol SH/mg protein)	14.7	15	17.1	17.2	14.5	1.69	0.12

^1^ Positive control (PC), negative control (NC), tributyrin (TRI-BUT), mono-, di-, triglycerides of butyric acid (MDT-BUT) and mono-, di-, triglycerides of lauric acid (MDT-LAU). ^2^ Different lowercase letters (a, b, c) on the same line indicate treatment effect, that is, difference between groups.

## Data Availability

The data presented in this study are available on request from the corresponding author. The data are not publicly available due an agreement between the authors.
